# Novel properties of photofermentative biohydrogen production by purple bacteria *Rhodobacter sphaeroides*: effects of protonophores and inhibitors of responsible enzymes

**DOI:** 10.1186/s12934-015-0324-3

**Published:** 2015-09-04

**Authors:** Lilit Gabrielyan, Harutyun Sargsyan, Armen Trchounian

**Affiliations:** Department of Microbiology & Microbes and Plants Biotechnology, Biology Faculty, Yerevan State University, 1 A. Manoukian Str., 0025 Yerevan, Armenia; Department of Biophysics, Biology Faculty, Yerevan State University, 1 A. Manoukian Str., 0025 Yerevan, Armenia

**Keywords:** *Rhodobacter sphaeroides*, Biohydrogen photoproduction, Protonophores, Nitrogenase and hydrogenase inhibitors, ATPase activity

## Abstract

**Background:**

Biohydrogen (H_2_) production by purple bacteria during photofermentation is a very promising way among biological H_2_ production methods. The effects of protonophores, carbonyl cyanide *m*-chlorophenylhydrazone (CCCP), 2,4-dinitrophenol (DNP), and inhibitors of enzymes, involved in H_2_ metabolism, metronidazole (Met), diphenyleneiodonium (DPI), and dimethylsulphoxide (DMSO) on H_2_ production by *Rhodobacter sphaeroides* MDC6522 isolated from Jermuk mineral springs in Armenia have been investigated in both nitrogen-limited and nitrogen-excess conditions.

**Results:**

With the increase of inhibitors concentrations H_2_ yield gradually decreased. The complete inhibition of H_2_ production was observed in the presence of DPI and CCCP. DPI’s solvent—DMSO in low concentration did not significantly affect H_2_ yield. *N,N′*-dicyclohexylcarbodiimide (DCCD)-inhibited the F_O_F_1_-ATPase activity of bacterial membrane vesicles was analyzed in the presence of inhibitors. Low concentrations of DPI and DMSO did not affect ATPase activity, whereas Met and CCCP stimulated enzyme activity. The effect of DNP was similar to CCCP.

**Conclusions and significance:**

The results have shown the low concentration or concentration dependent effects of protonophores and nitrogenase and hydrogenase inhibitors on photofermentative H_2_ production by *Rh. sphaeroides* in nitrogen-limited and nitrogen-excess conditions. They would be significant to understand novel properties in relationship between nitrogenase, hydrogenase and the F_O_F_1_-ATPase in *Rh. sphaeroides*, and regulatory pathways of photofermentation. The inhibitors of nitrogenase and hydrogenase can be used in biotechnology for regulation of H_2_ production in different technology conditions and development of scale-up applications, for biomass and energy production using purple bacterial cells.

## Background

**Fig. 1:**
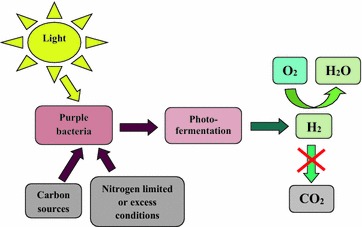
H_2_ production by purple bacteria during photofermentation of carbon sources and under light

Over the next 50 years biofuels [biohydrogen (H_2_), ethanol, and bio-methane] will be one of the most promising ways of energy supply. H_2_ is a very effective alternative energy source, because it produces high amount of energy (~140 MJ/kg), which is as least 3 times greater, than natural gas and hydrocarbon fuels [1–3]. H_2_ is considered as an environmentally friendly fuel, its combustion does not contribute the air contamination as there is no production of carbon dioxide, the only product of the reaction is water (Fig. 1) [1, 2, 4, 5]. Now, the world production of H_2_ is more than 50 million tons, and it is increased quickly over the world, it will decrease H_2_ production costs, which can be competitive with other fuels, such as oil and natural gas [3]. Thus, shortly H_2_ can become one of the main fuels in global energy economy.

In comparison with the traditional ways of H_2_ production such as thermochemical and photochemical processes, biological H_2_ production is known to be less energy intensive, because it carried out at ambient temperature and atmospheric pressure [2, 3]. H_2_ can be produced (1) by chemotroph bacteria during “dark” or “mixed-acid” fermentation of various carbon sources; (2) by microalgae and cyanobacteria during “direct” and “indirect” biophotolysis, resulting in water splitting, and (3) by purple bacteria during “photofermentation” of organic carbon substrates using sunlight as energy source (Fig. 1) [1–3, 6–8]. Among these, purple bacteria are highly favorable microorganisms for H_2_ production. Photosynthetic purple non-sulfur bacteria are able to produce H_2_ during photofermentation of organic carbon sources under anaerobic conditions using light as energy source [7–9]. As known, two types of enzymes—nitrogenase and hydrogenase are involved in photofermentative H_2_ metabolism in purple bacteria [2, 7, 8]. Purple non-sulfur bacterium *Rhodobacter sphaeroides* contains only one form of nitrogenase —[Mo–Fe]-nitrogenase [7, 8]; this is a binary enzyme, consisting of two metalloproteins: [Fe]-protein and [Mo–Fe]-protein [2, 10, 11]. These bacteria also contain [Ni–Fe]-hydrogenases, which are classified according to their involving in H_2_ metabolism: “H_2_-evolving”, “H_2_-uptake” and “bidirectional” hydrogenases [1, 11–13]. The latter can catalyse H_2_ uptake or production depending on the growth conditions.

During photofermentation purple bacteria can oxidize some organic carbon substrates to CO_2_, protons and electrons in tricarboxylic acid cycle (TCA) by generating NADH [7, 8]. The protons are pumped through the bacterial membrane during the photosynthetic electron transport with generation of proton motive force (Δ*p*). Under nitrogen-limited conditions or in the absence of N_2_, upon light H_2_ production by purple bacteria is mainly mediated by nitrogenase, which catalyzes conversion of protons to H_2_ by using energy from ATP, which is generated via the proton-translocating F_O_F_1_-ATPase; while hydrogenases in *Rh. sphaeroides* are usually involved in H_2_ uptake. But these hydrogenases can be reversible depending on the conditions: the reversibility of hydrogenases might be similar to the situation with hydrogenases in *Escherichia coli* or cyanobacteria [1, 3, 8, 13].

Carbonyl cyanide *m*-chlorophenylhydrazone (CCCP) and 2,4-dinitrophenol (DNP), protonophores functioning as uncouplers and dissipating Δ*p*, and metronidazole (Met), a low-range electron acceptor (redox potential (*E*_*h*_) is equal to −325 mV), have been shown to inhibit nitrogenase activity in cyanobacteria [14–20]. Diphenyleneiodonium (DPI) was established as an inhibitor of hydrogenase activity in *Rh. capsulatus* and *Chlamydomonas reinhardtii* [21, 22]. Dimethylsulphoxide (DMSO) (solvent of DPI) affected the bacterial growth properties and membrane stability [23, 24]. In our previous works, we demonstrated the inhibitory effects of high concentrations of DPI, DMSO and Met on H_2_ production by *Rh. sphaeroides* strain MDC6521 isolated from Arzni mineral springs in Armenia [25, 26]. The hydrogenase activity in *Rh. sphaeroides* and its relationship with nitrogenase and the F_O_F_1_-ATPase were suggested. Moreover, light and dark alternations affected H_2_ production by *Rh. sphaeroides* [9]. However, there are no data on effects of protonophores and those inhibitors at low concentrations on H_2_ production ability of *Rh. sphaeroides*. It is known, that many chemicals show biological effects at low and ultra-low concentrations [27, 28]. This is very interesting phenomenon; however, the mechanisms of low and ultra-low concentrations effects are not clear. It is interesting, how the effects of compounds used in low concentrations differ from those of relatively high concentrations and for other bacterial strains. Appropriate mechanisms of photofermentation and H_2_ production are still not clear; further studies will be needed.

In the present study we have investigated the effects of protonophores such as CCCP, DNP, inhibitors of nitrogenase and hydrogenase such as Met, DPI, and DMSO at different concentrations on H_2_ production ability depending on the nitrogen-limited and nitrogen-excess conditions in *Rh. sphaeroides* strain MDC6522, isolated from the other mineral springs in Armenian mountains—Jermuk. Novel and significant experimental data about the concentration-dependent effects of protonophores and inhibitors of nitrogenase and hydrogenase in *Rh. sphaeroides* have been obtained. The results would improve our understanding of mechanisms, regulatory pathways of bacterial H_2_ metabolism and bioenergetics of photofermentation. They can be helpful for determining the role of various enzymes and the interaction between them in H_2_ production depending on the growth conditions. Thus they might lead to optimization of the technology conditions for efficient H_2_ production. Importantly, the effects might be applied in H_2_ biotechnology, energy production using purple bacteria.

## Results

### Effect of various inhibitors on *Rh. sphaeroides* growth properties

Photofermentative H_2_ production by purple non-sulfur bacteria is known to be catalyzed by nitrogenase and hydrogenase. During the photosynthetic electron transport protons are pumped through the membrane with generation of Δ*p*, which is used to generate ATP via the F_O_F_1_-ATPase and to transfer electrons to ferredoxin (Fd). It is known that then Fd and ATP are used to generate H_2_ via nitrogenase [2, 7, 8]. As it was shown in our previous papers [25, 26], H_2_ production was strongly inhibited by high concentrations of DPI and Met. The effect of various compounds such as hydrogenase inhibitor DPI and its solvent DMSO, nitrogenase inhibitor Met, protonophore CCCP and their concentrations on growth peculiarities and photofermentative H_2_ production by *Rh. sphaeroides* strain MDC6522, in comparison with the other strain MDC6521, isolated from Arzni mineral springs, was studied.

**Fig. 2:**
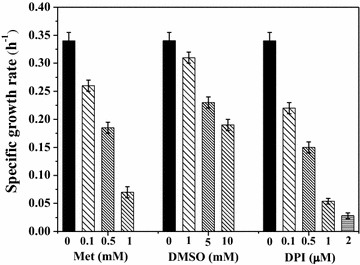
Specific growth rates of *Rh. sphaeroides* MDC6522 in batch culture in the presence of Met, DMSO and DPI various concentrations. Control was bacterial culture, grown in the medium without inhibitors

The growth properties were determined during anaerobic growth of *Rh. sphaeroides* MDC6522 upon illumination. The compounds used affected the specific growth rate of bacterial culture. Figure 2 shows a comparison of the growth specific rates of *Rh. sphaeroides*, grown in the presence of different inhibitors. With the increase of reagents concentrations, the specific growth rate gradually decreased. 1–2 µM DPI decreased the specific growth rate 6- to 12-folds (p < 0.001), whereas 1 mM Met suppressed growth rate ~5-fold (p < 0.001) (see Fig. 2). *Rh. sphaeroides* was unable to grow in the medium with CCCP and DNP, and both uncouplers were added after 24 h growth of culture, after then bacterial growth was strongly inhibited (not shown). The effect of DPI’s solvent DMSO on the culture specific growth rate was also studied for revealing the inhibitory effect of DPI. In culture with 1 mM DMSO this rate was 1.3-fold (p < 0.01) lower than that of the control, whereas 5–10 mM DMSO suppressed the specific growth rate ~1.5- to 2-folds (p < 0.01) in comparison with the control (Fig. 2). These data were similar to the results on DPI and DMSO effects obtained for the other strain MDC6521 [26], but *Rh. sphaeroides* MDC6521 was more sensitive to the inhibitors used.

### Effect of various inhibitors for enzymes on medium pH and *E*_*h*_ during *Rh. sphaeroides* anaerobic growth

**Fig. 3:**
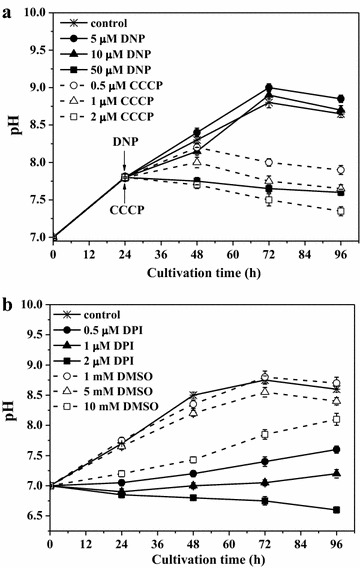
The effects of DNP and CCCP (**a**), DPI and DMSO (**b**) various concentrations on medium pH during *Rh. sphaeroides* MDC6522 anaerobic growth in batch culture upon illumination. DNP and CCCP were added after 24 h bacterial growth

The growth medium pH is an important parameter for bacterial growth under different conditions [9, 29–31]. During the anaerobic growth of *Rh. sphaeroides* MDC6522 control cells up to 72 h in nitrogen-limited anaerobic conditions, the pH of medium has risen from 7.0 ± 0.2 (initial pH) up to 8.8 (Fig. 3a). This increase can be caused by the carbon source utilization and OH^−^ ions efflux or by the polyhydroxybutyrate formation [29]. After then, during growth up to 96 h, pH decreased, which can be caused by the generation of photofermentation end-products, particularly acids, which could decay with H_2_ evaluation. Also during H_2_ generation the co-evolved CO_2_ can moderate pH change.

All inhibitors affected the pH of culture growth medium (Fig. 3). The pH value increased to ~9.0 by addition of 5–10 µM DNP (after 24 h growth) (Fig. 3a). The other kinetics of pH was observed by the addition of CCCP (after 24 h growth): pH increased to ~7.8–8.0 in the presence of 0.5–1 µM CCCP, whereas in the presence of 2 µM CCCP and 50 µM DNP pH of medium was not changed (see Fig. 3a). In the presence of 0.5–1 µM DPI the pH of medium was not changed much, and it decreased to ~6.7 in medium with 2 µM DPI (Fig. 3b). By addition of 1–5 mM DMSO pH changes during bacterial growth were similar to the control, and pH was not changed much in the presence of 10 mM DMSO (see Fig. 3b). In the presence of Met pH change during *Rh. sphaeroides* anaerobic growth was similar to the control (not shown).

**Fig. 4:**
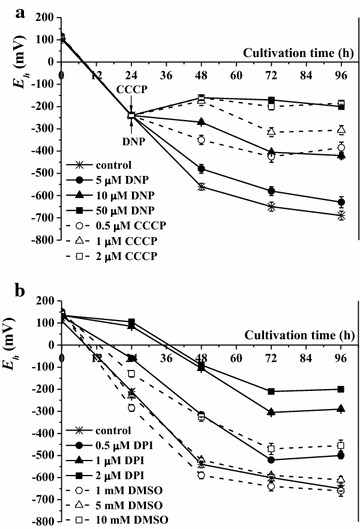
The effects of DNP and CCCP (**a**), DPI and DMSO (**b**) various concentrations on medium *E*_*h*_ during *Rh. sphaeroides* MDC6522 anaerobic growth in batch culture upon illumination. DNP and CCCP were added after 24 h bacterial growth

Redox potential *(E*_*h*_) is another significant parameter of the bacterial growth medium, which can be determined as the ability of a biological system to oxidize or reduce different substrates [9, 31–33]. According to the Nernst equation *E*_*h*_ depends on the reduced and oxidized products of fermentation, as well as on pH [29–31]. *E*_*h*_ of *Rh. sphaeroides* MDC6522 control cells decreased to −650 ± 20 mV during growth up to 72 h in nitrogen-limited anaerobic conditions (Fig. 4a). In the medium with 5 µM DNP *E*_*h*_ decreased to −580 ± 20 mV; whereas with 50 µM DNP *E*_*h*_ dropped to −170 ± 10 mV only (see Fig. 4a). In contrast to DNP, the other protonophore—CCCP delayed drop in *E*_*h*_. The inhibition of bacterial growth may arise from the effect of CCCP (1–2 µM) on *E*_*h*_, which was decreased to −315 ± 20 and −200 ± 15 mV, respectively, during 72 h culture growth (see Fig. 4a). In the medium with 0.5 mM Met *E*_*h*_ decreased to −410 ± 5 mV; whereas with 1 mM Met *E*_*h*_ dropped to −210 ± 10 mV only (not shown). Therefore, the used compounds affect the *E*_*h*_ in a concentration-dependent manner. Change of *E*_*h*_ can be caused by indirect effect of these reagents on *E*_*h*_, or by redox processes on a surface of bacterial membrane. Thus, the negative values of *E*_*h*_ and reduced medium are required for bacterial growth.

*E*_*h*_ of *Rh. sphaeroides* str. MDC6522 control cells, grown in nitrogen-excess anaerobic conditions up to 72 h, decreased to −600 ± 15 mV (Fig. 4b). The addition of DPI into the growth medium also affected *E*_*h*_: in the medium with 0.5 µM DPI *E*_*h*_ decreased to −520 ± 10 mV; whereas with 2 µM DPI *E*_*h*_ did not change much (Fig. 4b). 1 mM DMSO increased *E*_*h*_ up to −640 ± 20 mV. At the same time *E*_*h*_ gradually decreased during the growth from 5 to 10 mM DMSO: *E*_*h*_ drop was more intensive in the presence of 10 mM DMSO (up to −470 ± 25 mV) (see Fig. 4b).

### Effect of various inhibitors for enzymes on H_2_ photoproduction during *Rh. sphaeroides* anaerobic growth

The analysis of *E*_*h*_ changes gives information not only on main redox processes but also on H_2_ yield during bacterial anaerobic growth. There is a direct relationship between changes of *E*_*h*_ and H_2_ production by these bacteria; the reduction of protons to H_2_ is observed under strong reducing conditions [9, 31, 32, 34].

**Fig. 5:**
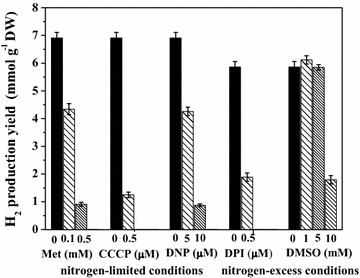
The effects of inhibitors various concentrations on H_2_ yield of *Rh. sphaeroides* MDC6522 during anaerobic growth in batch culture up to 72 h. The H_2_ yield was calculated by decrease in *E*_*h*_ (see “Methods”)

H_2_ yield of *Rh. sphaeroides* MDC6522 control cells during growth up to 72 h in nitrogen-limited anaerobic conditions was 6.91 mmol H_2_ (g dry weight (DW))^−1^, whereas in nitrogen-excess conditions H_2_ yield was ~1.2-fold lower (Fig. 5). As it can be seen from Fig. 5, four reagents used, except 1–5 mM DMSO, inhibited H_2_ production by *Rh. sphaeroides*. With the increase of inhibitors concentrations, the H_2_ yield gradually decreased. By addition of 0.1 and 0.5 mM Met H_2_ yield has decreased ~1.6-fold (p < 0.01) and ~7.6-fold (p < 0.001), respectively (see Fig. 5). The effect of Met on H_2_ yield might be coupled with change of photosynthetic electron transfer with Met as a preferred acceptor of electrons, instead of nitrogenase.

CCCP and DNP are two well-known protonophores, which are used to dissipate proton gradient responsible for ATP generation via the F_o_F_1_-ATPase and to uncouple photophosphorylation from photosynthetic electron transfer [35]. The Fig. 5 shows the H_2_ yield level in the presence of various concentrations of CCCP and DNP. In medium with 1–2 µM CCCP H_2_ production by bacterium has not been observed during 72 h growth, and has decreased ~5.5-fold (p < 0.001) in the presence of 0.5 µM CCCP (see Fig. 5). H_2_ yield lowered ~1.6-fold (p < 0.01) and ~8.0-fold (p < 0.001) in the medium with 5 and 10 µM DNP, and was not observed in the presence of 50 µM DNP. It is suggested that protonophores can decrease H_2_ generation by inhibiting of ATP synthesis by photophosphorylation, which is significant for nitrogenase-dependent photofermentative H_2_ production. These data were similar to the results obtained by Skizim and co-workers for cyanobacteria *Cyanothece* [35].

In medium with 1–2 µM DPI, the production of H_2_ by *Rh. sphaeroides* has not been observed during 72 h growth, and has decreased ~3.1-fold (p < 0.001) by addition of 0.5 µM DPI in comparison with the control (see Fig. 5). As shown in Fig. 5, DPI’s solvent—DMSO in concentrations of 1–5 mM did not affect H_2_ production by *Rh. sphaeroides*, whereas the high concentrations (10 mM) suppressed the H_2_ yield (~3.3-fold). These results were similar to those obtained for MDC6521, and they indicated that DMSO could inhibit H_2_ production depending on its concentration [26]. H_2_ production by the other strain—*Rh. sphaeroides* MDC6521 is more sensitive to the inhibitors action. DPI, DNP and CCCP irreversibly repress the H_2_ production; however DMSO and Met reversibly inhibit this process. When Met and DMSO was added into the growth medium, H_2_ started to be produced after 144–168 h (not shown) growth, probably, according to the recovery of enzyme activity.

### Effects of various inhibitors for enzymes on ATPase activity of *Rh. sphaeroides* membrane vesicles

**Fig. 6:**
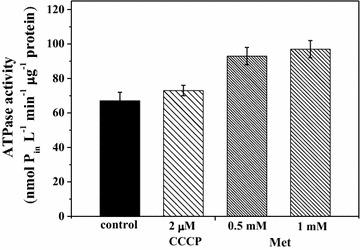
ATPase activity of *Rh. sphaeroides* MDC6522 membrane vesicles, which was calculated by colorimetric determination of liberation of inorganic phosphate (P_in_) per time and protein upon ATP adding (see “Methods”)

The F_O_F_1_-ATPase activity of *Rh. sphaeroides* MDC6522 membrane vesicles was analyzed in the presence of inhibitors to reveal the role of ATPase in H_2_ production. The F_O_F_1_-ATPase of purple bacteria belongs to F-type ATPase [36]. The membrane vesicles of bacteria, grown in the absence of inhibitors, demonstrated definite ATPase activity. By addition of 0.2 mM *N,N′*-dicyclohexylcarbodiimide (DCCD) ATPase activity was suppressed ~2-fold (p < 0.01) (not shown). Low concentrations of DPI and DMSO did not affect the enzyme activity, whereas CCCP (2 µM) stimulated ATPase activity on 10 % (Fig. 6). Similar data were obtained by the addition of DNP (not shown) and Met (0.5 and 1 mM), enhancing this enzyme activity on ~40–45 % (see Fig. 6). This effect can be attributed to the F_O_F_1_-ATPase, because DCCD specifically inhibits the F_O_F_1_-ATPase in various bacteria [31, 32]. It is possible, that these effects might be a result of inhibitors effect on ATPase via binding with enzyme and changing its activity, or on *E*_*h*_, which can regulate the F_O_F_1_-ATPase activity.

## Discussion

Purple non-sulfur bacteria are the most studied photosynthetic bacteria due to their demonstrated high H_2_ production yield. Two enzymes—nitrogenase and hydrogenase are involved in H_2_ metabolism in these bacteria. In the anaerobic nitrogen-limited conditions, during bacterial non-oxygenic photosynthesis, organic carbon sources are oxidized to CO_2_, protons and electrons. H^+^ can be recombined by a nitrogenase to produce H_2_ using energy of ATP, which is generated by the F_O_F_1_-ATPase during the work of photosynthetic apparatus [2, 7, 8]. The participation of hydrogenase in H_2_ production is also suggested under the other—nitrogen-excess conditions [1, 25, 26]. But appropriate mechanisms of photofermentation and H_2_ production depending on conditions are not clear yet. Thereby, two aspects of photofermentative H_2_ production by *Rh. sphaeroides* are interesting: 1st—type of enzyme (nitrogenase or hydrogenase), which is responsible for H_2_ production depending on the nitrogen-limited or nitrogen-excess conditions, and the relationship between these enzymes; and 2nd—a role of the F_O_F_1_-ATPase in photofermentation and H_2_ production by *Rh. sphaeroides*.

**Fig. 7:**
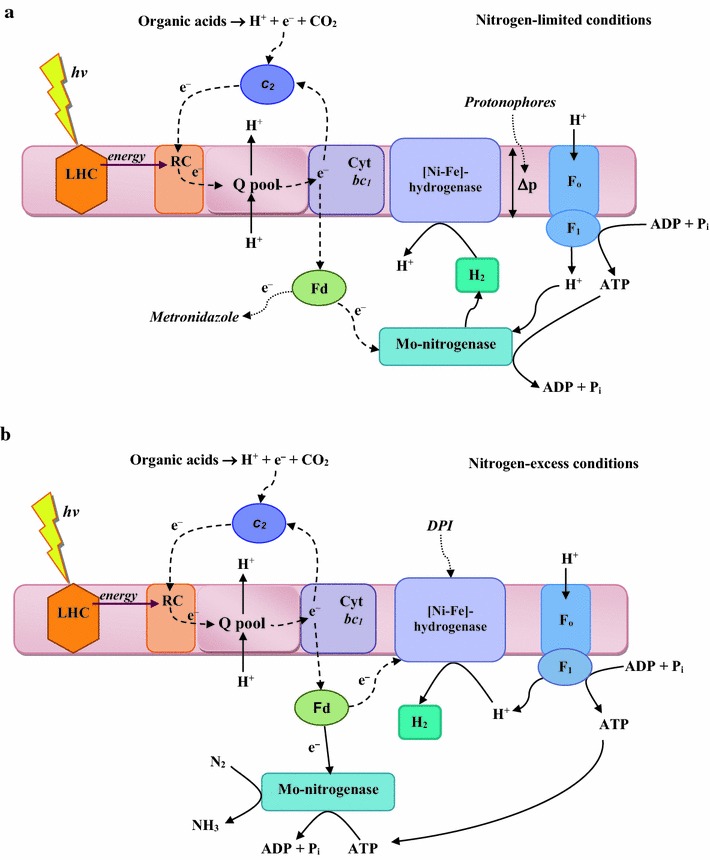
Proposed pathways involved in H_2_-metabolism in *Rh. sphaeroides* in nitrogen-limited (**a**) and nitrogen-excess (**b**) conditions. *LHC* light harvesting complex, *RC* reaction center, *Q* ubiquinone, *Fd* ferredoxin, *Cyt* cytochrome, *Δp* proton motive force, *P*_*i*_ inorganic phosphate. See the text

In this study the comparative analysis of protonophores and various inhibitors low concentrations effects on photofermentative H_2_ production during *Rh. sphaeroides* MDC6522 anaerobic growth in nitrogen-limited and nitrogen-excess conditions are presented. Two possible pathways of H_2_ generation in nitrogen-limited and nitrogen-excess conditions can be suggested (Fig. 7). H_2_ production from various carbon sources by purple bacteria is observed in anaerobic conditions under illumination (see Fig. 1). H_2_ yield of *Rh. sphaeroides* control cells during growth up to 72 h in nitrogen-excess anaerobic conditions was ~1.2-fold lower in comparison with nitrogen-limited conditions (see Fig. 5). In nitrogen-excess conditions nitrogenase catalyzes the reduction of N_2_ to ammonia according to the reaction: N_2_ + 8H^+^ + 8e^−^ + 16ATP → 2NH_3_ +H_2_ + 16ADP + 16P_i_, which leads to the generation of 1 mol H_2_ per mole of N_2_ fixed [4, 7, 8]. However, in nitrogen-limited conditions nitrogenase catalyzes reduction of protons to H_2_ according to the reaction: 8H^+^ + 8e^−^ + 16ATP → 4H_2_ + 16ADP + 16Pi. Thereby, in these conditions 4 times more H_2_ can be produced.

In the presence of protonophores and Met H_2_ yield has decreased ~5–8-folds. It is known, that CCCP and DNP are protonophores, which dissipate the Δ*p* and inhibit the synthesis of ATP via the F_O_F_1_-ATPase [14–17, 35]. Indeed, these protonophores can inhibit nitrogenase-dependent photofermentative H_2_ production by inhibiting the synthesis of ATP (Fig. 7a). The mechanisms of the Met inhibition are not clear yet, but it is known, that Met as a low-range electron acceptor can interact with the low potential electron carriers (Fd, flavodoxin) in photosynthetic electron transfer chain. The inhibitory effects of Met on the H_2_ yield in *Rh. sphaeroides* may be associated with dysfunction of the photosynthetic electron transport chain (Fig. 7a). Met penetrates into the bacterial cell through passive diffusion, where its nitro-group is reduced to reactive cytotoxic nitro-radicals by reduced Fd [37, 38]. Fd works as electron acceptors of nitrogenase, hydrogenase and other enzymes in anaerobic bacteria. It is known, that the selective toxicity of Met for anaerobic microorganisms is due to the redox potential of their electron transport components, which are sufficiently negative to reduce the nitro-group of Met [38].

Reversibility of hydrogenases is suggested for various chemotropic and phototrophic bacteria [1, 3, 12, 13, 39], so hydrogenase in *Rh. sphaeroides* might be bidirectional involved in H_2_ production (Fig. 7b). The complete inhibition of H_2_ production by *Rh. sphaeroides* was observed in the presence of 1–2 µM DPI, whereas DPI’s solvent—DMSO did not significantly affect H_2_ yield. The results with the inhibitory effects of DPI on H_2_ yield in *Rh. sphaeroides* provide a new evidence of involvement of hydrogenase in H_2_ production by these bacteria.

Then, to understand the role of ATPase in H_2_ production by *Rh. sphaeroides* the F_O_F_1_-ATPase activity of bacterial membrane vesicles was investigated. Low concentrations of DPI and DMSO did not affect the enzyme activity, whereas CCCP (2 µM) and Met (0.5–1 mM) enhanced ATPase activity on 10 % and ~40–45 %, respectively. These effects might be a result of inhibitors effect on ATPase via binding with the enzyme and changing its activity. Indeed, CCCP suppressed transfer of H^+^ by whole cells of *Rh. sphaeroides,* as shown before [40], which confirms the role of the Δ*p* in the activity of enzymes responsible for H^+^ transfer and H_2_ production.

Thus, two possible routes of H_2_ production by *Rh. sphaeroides* can be suggested (Fig. 7). Oxidation of organic acids generates electrons, which are passed through various photosynthetic electron transfer carriers to Fd, and protons, which are pumped through the membrane generating a Δ*p*. The latter derives the synthesis of ATP from ADP and inorganic phosphate (P_i_) via the F_O_F_1_-ATPase. Then Fd and ATP are used to generate H_2_ via nitrogenase. Protonophores used can inhibit nitrogenase-dependent H_2_ production by suppressing synthesis of ATP, whereas the Met can interact with Fd in photosynthetic electron transfer chain and can work as an alternative electron acceptor, instead of nitrogenase.

## Conclusions and significance

The data have shown low concentration or concentration dependent effects of protonophores and nitrogenise and hydrogenase inhibitors on photofermentative H_2_ production by *Rh. sphaeroides* in nitrogen-limited and nitrogen-excess conditions. The results obtained are significant to understand the relationship between nitrogenase, hydrogenase and the F_O_F_1_-ATPase and their roles during photofermentation and H_2_ production in *Rh. sphaeroides.* The relationship if any can be considered as a novel property of these enzymes. Importantly, the relationship depends on the nitrogen-excess and nitrogen-limited conditions. Thus, protonophores and nitrogenase and hydrogenase various inhibitors at different concentrations can be applied in the development of scale-up H_2_ production biotechnology, for biomass and energy production using purple bacterial cells.

## Methods

### Bacterial strain and growth conditions

In the present work we used *Rh. sphaeroides* strain MDC6522 (Microbial Depository Center, Armenia, WDCM803), which was isolated from Jermuk mineral waters (pH 6.5–8.5, 57–64 °C) in Armenian mountains [9, 30]. Bacteria were grown in batch culture anaerobically upon illumination (~36 W m^−2^) in Ormerod medium with succinate as a carbon source and yeast extract as a nitrogen source as described previously [9, 30, 31]. The growth of bacterial culture was recorded by changes in optical density (OD_660_) using a Spectro UV–Vis Auto spectrophotometer (Labomed, USA), and by determining DW of bacterial biomass, which was correlated with OD_660_ according the equation: DW (g L^−1^) = OD_660_ × 0.48. The specific growth rate was calculated, as described previously [9, 26, 31].

In order to create conditions of nitrogen source limitation, the media was supplied with yeast extract (2 g L^−1^); whereas to create nitrogen-excess conditions the concentration of yeast extract was increased 2.5-fold (the media was supplied with 5 g L^−1^ yeast extract). Yeast extracts contain various amino acids, vitamins and other growth stimulating compounds and therefore it can be used as a component of growth media for the cultivation of various microorganisms [30, 41].

The concentrations of DPI and CCCP added into the growth medium ranged from 0.5 to 2 µM; DNP—from 5 to 50 µM; Met—from 0.1 to 2 mM, DMSO—from 1 to 10 mM.

### Determinations of pH, *E*_*h*_ and H_2_ yield

The initial pH of the culture medium was maintained to 7.0 ± 0.1 by 0.1 M NaOH or 0.1 M HCl and determined at certain time intervals (0–96 h) by a pH-meter (HANNA Instruments, Portugal) with selective pH electrode, as described [9, 25, 31].

The medium *E*_*h*_ was determined during *Rh. sphaeroides* growth using a pair of redox (platinum (Pt) and titanium–silicate (Ti–Si)) and reference (Ag/AgCl) electrodes, as described before [9, 26, 31]. Note Ti–Si electrode measures the overall *E*_*h*_, whereas Pt electrode (sensitive to O_2_ and H_2_) under anaerobic conditions detects only H_2_ [30, 32]. *E*_*h*_ kinetics determined using redox electrodes during culture growth gives information about main redox processes and also H_2_ generation [31, 32]. The H_2_ yield was evaluated by the drop of *E*_*h*_ to low negative values using correlation between *E*_*h*_ change and H_2_ evolution and was expressed in mmol H_2_ (g DW)^−1^ [9, 31]:

$$ {\text{H}}_{ 2} {\text{ yield }} = \frac{{{\text{Amount of produced H}}_{ 2} {\text{ (mmol L}}^{ - 1} )}}{{{\text{Dry weight (g L}}^{ - 1} )}}. $$

This determination of H_2_ is close to the method with Clark-type electrode employed by other authors [42, 43]. H_2_ generation was confirmed by the chemical method, as described [26, 44].

### ATPase activity assay

ATPase activity of *Rh. sphaeroides* bacterial membrane vesicles was determined by the liberation of inorganic phosphate (P_in_) in the reaction with ATP by the spectrophotometric method, as described [31, 32], and it was expressed in nmol P_in_ per L per µg protein in 1 min. Membrane vesicles were prepared by the method, as described previously [26, 31]. For inhibitors effects studies, the membrane vesicles were incubated with inhibitors for 10 min.

### Reagents and data processing

CCCP, Met, DPI, DNP, DMSO, DCCD, ATP were obtained from Sigma, Aldrich (USA); yeast extract was purchased from Carl Roth GmbH (Germany) and succinic acid was obtained from Unichem (China). The other reagents of analytical grade were used in this study.

Each experiment was repeated three times to determine deviations, which are displayed as error bars on figures. The standard errors were calculated and Student criteria (p) were employed to validate the difference in average data between different series of experiments, as described previously [26, 31].

## Abbreviations

CCCP: carbonyl cyanide m-chlorophenylhydrazone
DCCD: *N,N’*-dicyclohexylcarbodiimide
DMSO: dimethylsulphoxide
DNP: 2,4-dinitrophenol
DPI: diphenyleneiodonium
DW: dry weight
*E*_*h*_: redox potential
Met: metronidazole
OD: optical density
TCA: tricarboxylic acid cycle
Δ*p*: proton motive force
